# Concomitant Intake of Quercetin with a Grain-Based Diet Acutely Lowers Postprandial Plasma Glucose and Lipid Concentrations in Pigs

**DOI:** 10.1155/2014/748742

**Published:** 2014-04-16

**Authors:** Silvia Wein, Siegfried Wolffram

**Affiliations:** Institute of Animal Nutrition and Physiology, Christian-Albrechts-University of Kiel, Hermann-Rodewald-Straße 9, 24118 Kiel, Germany

## Abstract

Treatment goals of diabetes mellitus type 2 (DMT2) include glycemic control and reduction of nonglycemic risk factors, for example, dyslipidemia. Quercetin, a plant-derived polyphenol, often discussed for possible antidiabetic effects, was investigated for acute postprandial glucose- and lipid-lowering effects in healthy growing pigs. Male pigs (*n* = 16, body weight = BW 25–30 kg) were fed flavonoid-poor grain-based meals without (GBM) or with quercetin (GBMQ). In a first experiment, postprandial plasma concentrations of glucose, nonesterified fatty acids (NEFA), and triacylglycerols were analyzed in 8 pigs receiving 500 g of either GBM or GBMQ (10 mg/kg BW) in a cross-over design. Blood samples were collected before, and up to 5 h every 30 min, as well as 6 and 8 h after the feeding. In the second experiment, 2 h after ingestions of 1000 g of either GBM or GBMQ (50 mg/kg BW) animals were sacrificed; gastric content was collected and analyzed for dry matter content. Quercetin ingestion reduced postprandial glucose, NEFA, and TG concentration, but two hours after ingestion of the meal no effect on gastric emptying was observed. Our results point to inhibitory effects of quercetin on nutrient absorption, which appear not to be attributable to delayed gastric emptying.

## 1. Introduction

Diabetes mellitus type 2 (DMT2) is a chronic disease, characterized by peripheral insulin resistance and hyperglycemia [1]. The incidence of DMT2 has increased dramatically in the last decades, and for the year 2030 a worldwide increase up to 370 million diabetic individuals is estimated [2]. Treatment goals of DMT2 include adequate glycemic control and reduction of nonglycemic risk factors such as dyslipidemia [3].

Postprandial dyslipidemia is complex and involves, aside from disturbed fatty acid metabolism, a variety of factors including hyperinsulinemia, insulin resistance, and hyperglycemia [4]. Insulin resistance can cause postprandial dyslipidemia by increasing the enterocytic production of chylomicrons and by an impaired clearance capacity [4]. Postprandial dyslipidemia in turn seems to enhance endothelial dysfunction, oxidative stress, and inflammation [5]. High concentrations of nonesterified fatty acids (NEFA) inhibit insulin-dependent skeletal glucose uptake and insulin-mediated suppression of hepatic gluconeogenesis [6]. Thus, therapeutic strategies target postprandial dyslipidemia [7] as well as postprandial glucose response to improve overall glycemic control [8].

In addition to synthetic pharmaceuticals, the use of plants or plant extracts for the prevention and treatment of diseases has greatly increased [9]. In recent years, biological effects of flavonoids have been intensively investigated. With respect to energy metabolism and the sequel of metabolic disturbances eventually leading to insulin resistance and DMT2, quercetin was found to inhibit the intestinal glucose transport via glucose transporter 2 (GLUT 2) [10], to improve glucose uptake in adipocytes [11], to inhibit adipocyte differentiation [11], and to possess anti-inflammatory and antioxidative properties [12]. However, most results are derived from* in vitro* investigations and need further validation in the intact organism.

To investigate acute effects of orally administrated quercetin on postprandial glucose, NEFA, and triacylglycerol (TG) plasma concentrations, we applied quercetin to healthy growing pigs within a grain-based meal.

## 2. Materials and Methods

### 2.1. Chemicals

Quercetin (>98%) was obtained from Roth, Karlsruhe, Germany. All chemicals and solvents for high-performance liquid chromatography (HPLC) analysis were obtained from Across Organics Geel, Belgium, and were of HPLC grade.

Plasma TG, NEFA, and glucose were quantified spectrophotometrically using commercially available kits (TG and glucose: Thermo Clinical Labsystems, Vantaa, Finland, and FFA: Randox, Crumlin, UK).

### 2.2. Animals

All animal experiments were approved by Ministry of Agriculture, Environment and Rural Areas of Land Schleswig-Holstein, Germany, number V312-72241.121-25 (81-7/06). Pigs (*n* = 16, cross-bred, male, castrated pigs obtained from the Institute of Animal Breeding of the University of Kiel, body weight 25–30 kg) were surgically equipped with indwelling catheters (Cook Deutschland GmbH) placed into the jugular vein. They were fed 500 g of a commercial pig diet based on ground barley, wheat, rye, and defatted soy bean meal (per kg diet: 165 g crude protein, 21 g crude fat, 53 g crude fiber, 12.6 MJ metabolizable energy, and 897 g dry matter) without detectable levels of quercetin twice daily. Water was freely available by nipple drinkers.

#### 2.2.1. Postprandial Nutrient Absorption

To investigate the impact of quercetin on postprandial plasma concentrations of glucose, NEFA, and TG, 8 pigs received either the grain-based meal or the grain-based meal supplemented with quercetin (10 mg/kg body weight) in a cross-over design. Wash-out period between treatments was two days. On the days of the experiment, 12 h after their last meal, quercetin was mixed into the regular morning meal and fed immediately. Ingestion of these meals was completed within 3 minutes and water was freely available by nipple drinkers during the experiment. Blood samples were collected in the 12 h fasted state, and up to 5 h every 30 min, as well as 6 and 8 h after administration. Samples were drawn into heparinized blood containers and immediately centrifuged (1500 ×g, 10 min, 4°C). The plasma samples were stored at −80°C until analysis.

#### 2.2.2. Gastric Emptying

Gastric emptying was investigated in 8 pigs which were randomly assigned to one of two groups. Four pigs received either the grain-based meal (1000 g) or the grain-based meal supplemented with quercetin (50 mg/kg BW). On the day of the experiment, 12 h after their last meal, diet was mixed with water (1 : 2.5, w/v) 15 min before feeding. Thereafter quercetin was mixed into the meal of four animals and meals were immediately fed. Ingestion of these meals was completed within 10 min and blood samples were collected after 2 hours. After the blood sampling, animals were euthanized by injection of pentobarbital (0.3 mL/kg body weight release, WDT, Garbsen, Germany) into the jugular vein. Subsequently, stomachs were ligated at the gastroesophageal and pyloroduodenal junctions and removed from the bodies. Gastric content was completely removed, weighed, and analyzed for dry matter content according to Naumann et al. [13]. Blood samples were analyzed for total flavonol concentrations (quercetin + kaempferol + isorhamnetin + tamarixetin).

#### 2.2.3. High-Performance Liquid Chromatography Analysis of Flavonols

Flavonols were extracted from plasma according to Kumazawa et al. [12], and high-performance liquid chromatography analysis of flavonols was performed as described previously [5]. Shortly, an aliquot (980 mL) of the plasma sample was spiked with 20 mL of rhamnetin (internal standard, 1 mg/20 mL in methanol), acidified (pH 5) with 130 mL of acetic acid (0.583 mol/L), and subsequently treated with 75 mL of a mixture of b-glucuronidase and sulfatase (Sigma-Aldrich Chemie GmbH, Hamburg, Germany; type H-1, final activities: 7.300 and 130 U/mL glucuronidase and sulfatase, resp.). After incubation for 1 hour at 37°C, 3 mL of acetone was added, and the sample was centrifuged at 4°C at 3700 ×g for 45 minutes. The supernatant was evaporated until dryness (partial vacuum at 45°C, SPD2010 SpeedVac System, Thermo Fisher Scientific GmbH, Dreieich, Germany). Residues were resolved in 200 mL of methanol, 77.5 mL of nanopure H_2_O, and 22.5 mL of HCl (10 mol/L). Thirty microliters of the final solution was injected by a cooled (4°C) autosampler (Jasco, Groß-Umstadt, Germany) onto a C-18 Kromasil 100 column (Jasco, Groß-Q6 Umstadt, Germany) (250 × 4 mm, particle size 5 mm) guarded by a C-18 Inertsil ODS-2 precolumn (Jasco, Groß-Q6 Umstadt, Germany) placed in a column oven at 30°C. The eluent (flux rate 1 mL/min) was composed of 0.025 mol/L NaH_2_PO_4_, pH 2.4, acetonitrile, and methanol (68 : 27 : 5 v/v/v). For postcolumn derivatization, the effluent was mixed with Al(NO_3_)_3_ (1 mmol/L in methanol containing 7.5% [v/v] acetic acid) (flux rate 0.4 mL/min) in a postcolumn reactor. The fluorescence of the flavonol/aluminum complex was measured using a fluorescence detector (excitation wavelength: 422 nm, emission wavelength: 485 nm; FP920, Jasco). Detection limits for quercetin, isorhamnetin, and tamarixetin were 5–7 nmol/L. Interanalysis and interday variances were within 5%. Standards were prepared with pure flavonols (Carl Roth GmbH, Karlsruhe, Germany) and treated like samples. Identification of peaks obtained in plasma samples was performed using the retention times of the pure flavonols (standards).

### 2.3. Statistical Analysis

For calculations of area under the plasma concentration-time curves (AUC) the linear trapezoidal rule was used (AUC_0→8_). Data are presented as group means ± standard deviation (SD). Statistical analysis was performed using two-tailed paired* t*-test; level for significance was set, *P* < 0.05. All calculations were performed using GraphPad Prism (GraphPad Software Inc., Version 4.01, 2004, San Diego, USA).

## 3. Results and Discussion

### 3.1. Results

#### 3.1.1. Body Weight of Pigs Differed at No Time between the Treatment Groups

**Figure 1:**
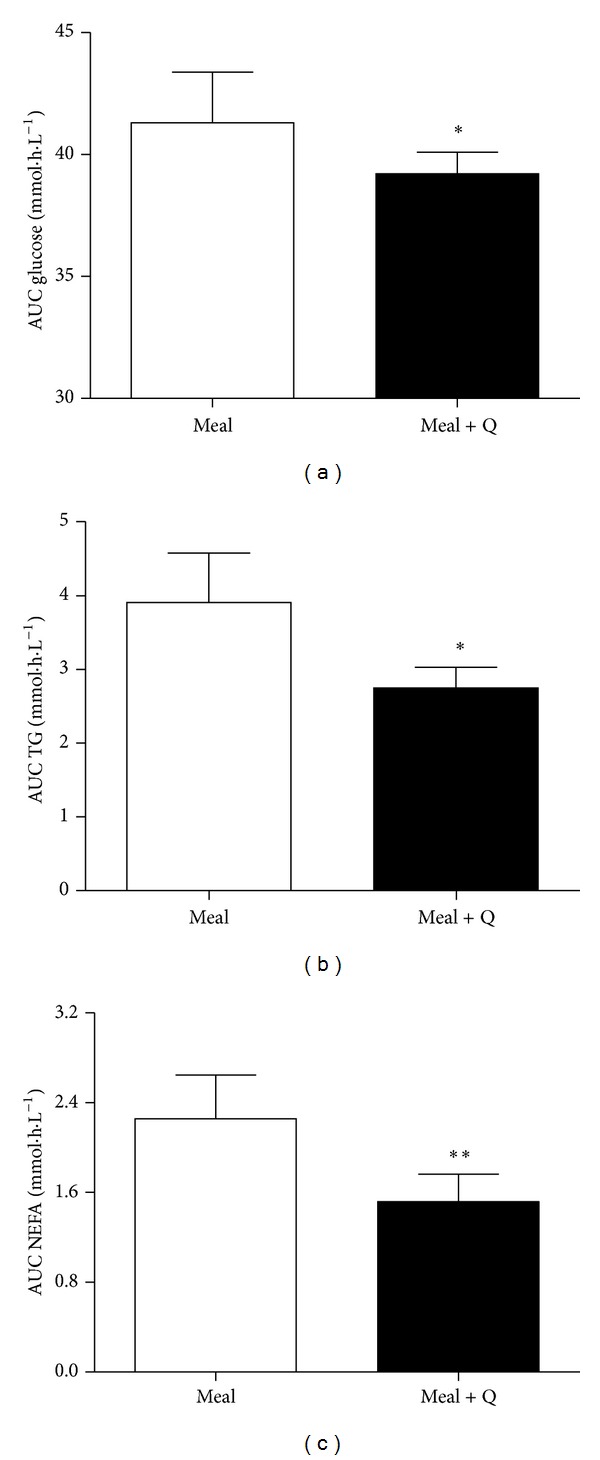
Effect of quercetin on postprandial concentrations of glucose, triacylglycerol, and nonesterified fatty acids. Area under the postprandial plasma concentration-time curves of glucose (a), triacylglycerol (b), and nonesterified fatty acids (c) after overnight (12 h) fasting followed by the ingestion of 500 g of a grain-based meal without (clear symbols) or with (filled symbols) the addition of quercetin (10 mg/kg body weight). Data are means (SD); paired, two-tailed* t*-test (*n* = 8), significant differences: ∗ indicates *P* = 0.01 and ∗∗ indicates *P* = 0.0026. AUC: area under the plasma concentration-time curve; NEFA: nonesterified fatty acids; TG: triacylglycerol.

*Postprandial Nutrient Absorption.* Fasted plasma glucose, triacylglycerol, and NEFA concentrations were not different between animals (Table 1) at both days of experiments. The postprandial glucose concentrations were lower when the grain-based meal was ingested concomitantly with quercetin (10 mg/kg body weight) as described by lower AUC (Figure 1(a)). Additionally we observed a reduction in postprandial plasma TG and NEFA concentrations when quercetin was ingested with the meal (Figures 1(b) and 1(c)).

**Figure 2:**
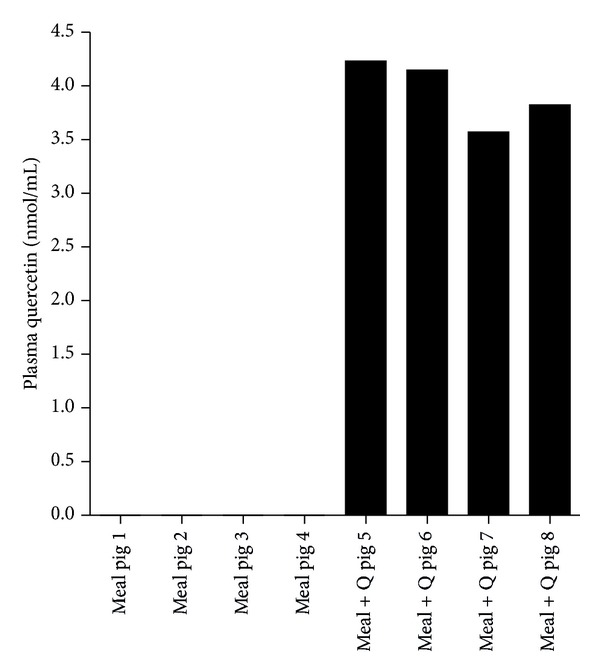
Plasma quercetin concentrations after the ingestion of grain-based meals with or without quercetin supplementation. Plasma quercetin concentrations measured 2 h after the intake of 1000 g of a grain-based meal without (clear symbols) or with (filled symbols) the addition of quercetin (50 mg/kg body weight) after enzymatic treatment of plasma samples (*n* = 8).

**Figure 3:**
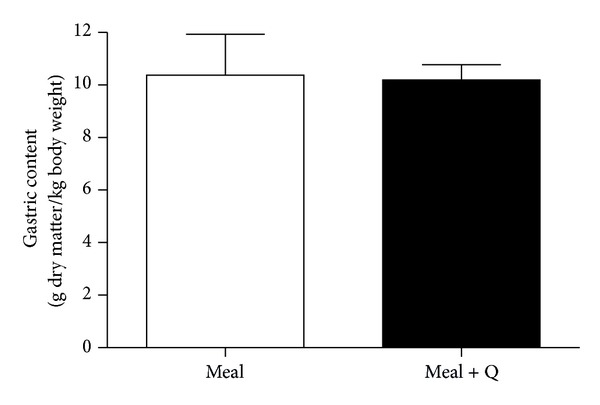
Gastric content after the ingestion of grain-based meals with or without quercetin supplementation. Dry matter content of gastric content 2 h after the ingestion of 1000 g of a grain-based meal without (clear symbols) or with (filled symbols) the addition of quercetin (50 mg/kg body weight). Paired, two-tailed* t*-test, *P* = 0.8767 (*n* = 8).

*Gastric Emptying.* At 2 h after ingestion of a grain-based meal with or without the addition of quercetin (50 mg/kg body weight) quercetin was detected (3.9 ± 0.3 nmol/mL) only in plasma of pigs fed with quercetin (Figure 2). The gastric emptying, however, was not affected by the ingestion of quercetin (Figure 3).

### 3.2. Discussion

Flavonoids, which are widely distributed in higher plants, have long been recognized for health promoting effects [14]. Although epidemiological studies point to an association between flavonoid intake and a reduced risk for certain chronic diseases including DMT2 [15], there is not much concise data on* in vivo* efficacy for many preparations [16–19]. Major challenges in evaluating the systemic bioactivity of flavonoids are due to differences deriving from the fate of flavonoids within the gastrointestinal tract such as sugar moiety, vehicle of application, food matrix effects, and the species-specific metabolism [20]. After ingestion, quercetin is subjected to first-pass metabolism in enterocytes of the small intestine and the liver (conjugation reactions including sulfation, glucuronidation, and methylation) [19] resulting in species-specific metabolites. Thus nonconjugated quercetin is virtually absent in rats [21], pigs [22], and humans [23], rather low in dogs (16% of metabolites with intact flavonol structure) [24], and comparably high in horses (47% of metabolites with intact flavonol structure) [25]. Although in humans, rats, and pigs glucuronidation is considered to be the major conjugation pathway, the conjugation patterns differ. While in pigs and humans only 20–40% of total flavonols occur in methylated form, this fraction is the major metabolite (70%) found in rats [23, 24]. Thus, to draw conclusions from a model organism to human metabolism, the pig seems to be particularly suitable to investigate effects of quercetin. However, aside from systemic bioactivity, local intestinal effects of quercetin seem feasible [26].

The present study clearly shows that quercetin (10 mg/kg body weight) ingested at a low dose concomitantly with a grain-based meal reduced postprandial glucose plasma concentrations. The reduction in postprandial glucose concentration documented in this study is in line with a study where quercetin significantly decreased incremental plasma glucose after a single oral dose of starch and the area under the postprandial glucose response compared with the control group in streptozotocin-induced diabetic rats [27]. Although the present study did not examine intestinal glucose transport, mechanisms responsible for this effect are quite likely through *α*-glucosidase (EC 3.2.1.20) inhibition.* In vitro*, quercetin was shown to be a potent inhibitor of *α*-glucosidase [28] which was even more potent than acarbose, an *α*-glucosidase inhibitor extensively investigated and widely prescribed antidiabetic drug [29]. Inhibition of intestinal GLUT 2 is also conceivable [10]; either at the basolateral membrane of the enterocyte after passive diffusion of quercetin or luminal at the apical membrane of the enterocyte where GLUT 2 is transiently insertet during a meal [30]. Inhibition of the sodium-dependent glucose transporter 1 (SGLT1) by quercetin aglycone, however, can largely be ruled out [10, 31, 32]. Thus, quercetin aglycone might act as a potent luminal inhibitor of glucose absorption independent of its own transport.

However, aside from inhibited intestinal glucose uptake, lower postprandial glucose concentrations might have resulted from delayed gastric emptying. A study in mice indicated that the intraperitoneal injection of quercetin-rich extracts (30 and 100 mg/kg body weight) inhibited gastric emptying dose dependently [33]. In the present study, the concomitant ingestion of quercetin with a grain-based diet (50 mg/kg body weight) had no effect on gastric emptying at two hours after ingestion, a time point at which quercetin was already present in circulation.

With respect to its antidiabetic properties, quercetin might be even superior to sole *α*-glucosidase inhibitors. While they are known to effectively control postprandial glycemia [34–38], lipid-lowering effects are discussed controversially. Some authors report lipid-lowering activities [34, 36], whereas others found no such effects [35, 37, 38]. Quercetin has previously been shown to have lipid-lowering effects in rats [39, 40], pigs [41], and humans [42] when fed chronically. However, the present study reveals that quercetin exerts also acute lipid-lowering effects such as reduced postprandial plasma TG and NEFA concentrations. Although we cannot determine the underlying mechanisms for these effects from the present study, it can be speculated that the inhibition of pancreatic lipase, the major enzyme responsible for dietary triglyceride digestion, may be included [43].

## 4. Conclusions

This study emphasizes glucose-lowering effects of quercetin after ingestion of a grain-based meal, which are less clear or even invisible when tested within a glucose challenge. In this context the observed effects of quercetin appear not to be attributable to delayed gastric emptying. Thus, quercetin ingestion might be suitable to acutely assist postprandial glucose and lipid control.

## Figures and Tables

**Table 1:** Fasted (12 h) plasma concentrations of glucose, triacylglycerol, and nonesterified fatty acids in pigs (*n* = 8).

Group	Glucose [mmol/L]	Triacylglycerol [mmol/L]	NEFA [mmol/L]
Meal	4.7 ± 0.5	0.38 ± 0.1	0.13 ± 0.06
Meal + quercetin	4.7 ± 0.6	0.36 ± 0.1	0.15 ± 0.09
